# Supplementary material for: Cervical and Vaginal Microbiomes in Early Miscarriages and Ongoing Pregnancy with and without Dydrogesterone Usage
Source: Int J Mol Sci. 2023 Sep 8;24(18):13836. doi: 10.3390/ijms241813836 (PMC10531357; doi:10.3390/ijms241813836)
Supplement: Supplementary file 1 [file ijms-24-13836-s001.zip › Table S1. CSTs in cervical microbiome (Fisher’s exact test).pdf]

Supplementary Table 1. CSTs in cervical microbiome (Fisher's exact test)

CSTs in cervical microbiome	Group I (ongoing pregnancy without progesterone, n=23)	Group II (ongoing pregnancy with progesterone, n=17)	Group III (miscarriages, n=11)	P I-II	P I-III	P II-III
CST I	-	-	-	-	-	-
CST II	-	-	-	-	-	-
CST III	18	12	8	0.72	1.00	1.00
CST IV, including<br>CST IVB<br>CST IVC0<br>CST IVC3	5<br>4<br>-<br>1	3<br>1<br>1<br>1	2<br>2<br>-<br>-	1.00<br>0.37<br>0.42<br>1.00	1.00<br>1.00<br>1.00<br>1.00	1.00<br>0.54<br>1.00<br>1.00
Unidentified	-	2 (Ileibacterium valens, Brochotrix thermosphacta)	1 (Mycoplasma girerdii)	0.17	0.32	1.00
**Total**	23	17	11			
