# Supplementary material for: Cervical and Vaginal Microbiomes in Early Miscarriages and Ongoing Pregnancy with and without Dydrogesterone Usage
Source: Int J Mol Sci. 2023 Sep 8;24(18):13836. doi: 10.3390/ijms241813836 (PMC10531357; doi:10.3390/ijms241813836)
Supplement: Supplementary file 1 [file ijms-24-13836-s001.zip › Table S2. CSTs in vaginal microbiome (Fisher’s exact test).pdf]

Supplementary Table 2. CSTs in vaginal microbiome (Fisher's exact test)

CSTs in vaginal microbiome	Group I (ongoing pregnancy without progesterone, n=23)	Group II (ongoing pregnancy with progesterone, n=17)	Group III (miscarriages, n=11)	P I-II	P I-III	P II-III
CST I	-	-	-	-	-	-
CST II	-	1	-	0.42	1.00	1.00
CST III	17	12	6	1.00	0.43	0.44
CST IV, including<br>CST IVB<br>CST IVC0<br>CST IVC3	6<br>5<br>-<br>1	2<br>1<br>-<br>1	3<br>3<br>-<br>-	0.43<br>0.22<br>-<br>1.00	1.00<br>1.00<br>-<br>1.00	0.35<br>0.27<br>-<br>1.00
Unidentified	-	2 (Ileibacterium valens)	2 (Mycoplasma girerdii, Bacteroides plebeius)	0.17	0.10	1.00
**Total**	23	17	11			
